# Supplementary material for: Brain Proteome-Wide Association Study Identifies Candidate Genes that Regulate Protein Abundance Associated with Post-Traumatic Stress Disorder
Source: Genes (Basel). 2022 Jul 27;13(8):1341. doi: 10.3390/genes13081341 (PMC9332745; doi:10.3390/genes13081341)
Supplement: Supplementary file 1 [file genes-13-01341-s001.zip › genes-1639995-SI.pdf]

The Supporting Information

Appendix: Table supplement

Introduction

A: Table S1. Supplement to the results of TWAS analysis in PTSD GWAS dataset

B: Table S2. Supplement to the results of overlapping gene brain imaging analysis

A:

Table 1: Supplement to the results of TWAS analysis in PTSD GWAS dataset

**Group**	**Population**	**CHR**	**SNP**	**Genes**	**Z-score**	***P*-value**
Rnaseq	All	2	rs7557285	*FBXO41*	3.44	9.85×10^{-6}
		19	rs2617688	*ZNF813*	3.66	3.485×10^{-4}
		1	rs949571	*ZBTB41*	-2.11	1.545×10^{-3}
		17	rs4985762	*FLCN*	2.80	1.80×10^{-3}
		12	rs7131767	*RERG*	2.93	3.60×10^{-3}
		1	rs34001546	*C1orf54*	-3.09	4.28×10^{-3}
		1	rs2936033	*TAF1A*	2.88	4.74×10^{-3}
		16	rs3091404	*ANKS4B*	-3.94	5.06×10^{-3}
		5	rs11747154	*PCDHA9*	-2.27	5.09×10^{-3}
		6	rs9477555	*KIF13A*	4.32	5.55×10^{-3}
		14	rs4905879	*HHIPL1*	-2.29	6.53×10^{-3}
		19	rs2927743	*ZNF793*	-2.59	8.86×10^{-3}
	Female	16	rs2377058	*CHMP1A*	-2.99	5.09×10^{-5}
		5	rs6879760	*PCDHA9*	-2.69	4.17×10^{-4}
		10	rs36014540	*RHOBTB1*	2.71	1.10×10^{-3}
		19	rs1529329	*ZNF417*	-3.30	1.17×10^{-3}
		6	rs1049346	*GLO1*	2.50	1.41×10^{-3}
		8	rs7007552	*LRRC69*	3.14	1.43×10^{-3}
		22	rs4474965	*DGCR8*	3.76	2.33×10^{-3}
		3	rs17050540	*RPUSD3*	2.04	2.50×10^{-3}
		1	rs4661055	*GPATCH4*	2.59	3.42×10^{-3}
		1	rs34001546	*C1orf54*	-2.76	5.15×10^{-3}
		11	rs3751097	*DDB2*	1.99	5.20×10^{-3}
	Male	3	rs2267846	*P4HTM*	2.13	1.82×10^{-43}
		10	rs4304660	*LOC100499489*	-2.71	1.16×10^{-6}
		1	rs6690515	*ATAD3A*	-4.10	2.59×10^{-5}
		10	rs4642993	*PRLHR*	-3.73	5.24×10^{-4}

		8	rs4876218	*CLN8*	3.04	5.37×10^{-4}
		10	rs749062	*OPTN*	-2.96	2.06×10^{-3}
		4	rs4696175	*TRIM2*	-3.13	2.87×10^{-3}
		1	rs1543294	*PMF1-BGLAP*	-2.86	4.00×10^{-3}
		11	rs7930356	*PGM2L1*	-3.51	4.05×10^{-3}
		19	rs383547	*RAB3D*	-2.43	6.75×10^{-3}
		1	rs7536099	*HHLA3*	-2.99	7.20×10^{-3}
Splicing	All	7	rs7798233	*AKAP9*	2.51	1.51×10^{-13}
		5	rs17564079	*POC5*	-2.30	1.11×10^{-7}
		3	rs11128490	*ZNF717*	3.05	1.55×10^{-5}
		7	rs741664	*PLXNA4*	3.69	1.23×10^{-4}
		2	rs3906948	*FAHD2B*	-2.78	2.88×10^{-4}
		3	rs1770638	*LSG1*	3.34	5.46×10^{-4}
		2	rs2084713	*UNC50*	2.20	6.04×10^{-4}
		5	rs392088	*BRD9*	3.25	1.53×10^{-3}
		11	rs308350	*NDUFV1*	-1.97	1.60×10^{-3}
		5	rs2240791	*SLC6A7*	2.89	1.77×10^{-3}
		6	rs3130557	*FLOT1*	3.21	2.07×10^{-3}
		5	rs7700814	*GLRX*	-2.42	2.31×10^{-3}
		17	rs16959820	*CCDC144A*	3.23	2.55×10^{-3}
		6	rs4712047	*SIRT5*	2.64	4.81×10^{-3}
		17	rs2241886	*GAA*	2.68	8.25×10^{-3}
		10	rs471917	*PHYH*	2.44	8.84×10^{-3}
		15	rs17392679	*SECISBP2L*	-2.80	9.78×10^{-3}
		11	rs17787912	*MADD*	-2.43	1.23×10^{-2}
		4	rs6855396	*C4orf21*	-2.27	1.38×10^{-2}
		19	rs892117	*MAP3K10*	-2.42	1.77×10^{-2}
		1	rs17035443	*SARS*	2.38	3.20×10^{-2}
		3	rs1836688	*MLF1*	2.24	3.40×10^{-2}
		8	rs17817071	*RIMS2*	-2.64	3.84×10^{-2}
	Female	2	rs6728452	*ZRANB3*	-2.48	3.78×10^{-8}
		2	rs11681740	*COL5A2*	-2.201	1.69×10^{-6}
		5	rs891943	*SLC6A7*	3.63	4.47×10^{-4}
		11	rs4757650	*LDHA*	-3.19	1.50×10^{-3}
		12	rs1147088	*GNS*	4.18	1.78×10^{-3}
		17	rs2010838	*AMZ2*	3.28	1.92×10^{-3}
		5	rs7700814	*GLRX*	-3.02	2.30×10^{-3}
		10	rs12247439	*STK32C*	2.88	3.55×10^{-3}

	7	rs6965194	*SGCE*	-3.08	5.69×10^{-3}
	7	rs13229505	*AKAP9*	2.57	1.19×10^{-10}
	2	rs17504837	*UNC50*	2.65	8.88×10^{-8}
	5	rs17564079	*POC5*	-2.31	6.55×10^{-7}
	7	rs741664	*PLXNA4*	4.15	4.56×10^{-5}
	19	rs17211813	*FKBP8*	3.45	3.23×10^{-4}
	2	rs17829296	*DNAH7*	-2.43	3.47×10^{-4}
	7	rs38417	*GGCT*	2.91	5.55×10^{-4}
	17	rs7221221	*CCDC144A*	3.39	7.14×10^{-4}
	7	rs6963510	*NSUN5P1*	3.16	8.36×10^{-4}
	6	rs4130023	*CCND3*	3.66	1.25×10^{-3}
Male	12	rs7315707	*STX2*	-3.51	1.30×10^{-3}
	4	rs7375984	*FSTL5*	3.53	3.52×10^{-3}
	4	rs2714841	*KLHL2*	2.93	3.55×10^{-3}
	1	rs1543294	*PMF1*	2.86	3.69×10^{-3}
	11	rs1458453	*SESN3*	-2.86	4.26×10^{-3}
	14	rs950388	*EML5*	-3.41	5.08×10^{-3}
	12	rs10842779	*ITPR2*	-3.11	7.62×10^{-3}
	8	rs12541532	*DENND3*	2.23	1.46×10^{-2}
	15	rs4287512	*ANKDD1A*	-2.67	1.81×10^{-2}
	5	rs4976210	*GTF2H2*	-2.09	2.96×10^{-2}
	5	rs4976210	*NAIP*	2.09	3.21×10^{-2}

Abbreviations: CHR, Chromosome; SNP, Single nucleotide polymorphism; *P*-value, Analytical permutation *P*-values.

B:

Table 2: Supplement to the results of overlapping gene brain imaging analysis

Gene	Phenotype ID	Brief description	*P*-value
MADD	1528	Mean MO in pontine crossing tract on FA skeleton	5.8×10^{-12}
	1442	Median T2star in left putamen	1.1×10^{-11}
	1443	Median T2star in right putamen	3.6×10^{-10}
	0198	Volume of Pallidum in the left hemisphere	8.7×10^{-10}
	0215	Volume of Pallidum in the right hemisphere	9.9×10^{-10}
	2863	Functional connectivity, connection 435 dimensionality 100	5.9×10^{-9}
	1349	Mean intensity of Accumbens-area in the left hemisphere	1.2×10^{-8}
	0371	Volume of superiorparietal in the left hemisphere	1.3×10^{-8}
	1880	Weighted-mean L3 in tract right cingulate gyrus part of cingulum	1.3×10^{-8}
	1638	Mean MD in cingulum hippocampus on FA skeleton (right)	1.3×10^{-8}
	0555	Volume of S-intrapariet+P-trans in the left hemisphere	1.6×10^{-8}
	1918	Mean ICVF in anterior limb of internal capsule on FA skeleton (right)	1.6×10^{-8}
	2122	Weighted-mean ISOVF (isotropic or free water volume fraction) in tract right superior longitudinal fasciculus	1.9×10^{-8}
	0464	Volume of superiorparietal in the left hemisphere	2.1×10^{-8}
	1444	Median T2star in left pallidum	2.4×10^{-8}
	1441	Median T2star in right caudate	2.4×10^{-8}
	0526	Volume of G-parietal-sup in the left hemisphere	4.0×10^{-8}
	1363	Mean intensity of Accumbens-area in the right hemisphere	4.4×10^{-8}
	1445	Median T2star in right pallidum	4.5×10^{-8}
GLO1	2936	Functional connectivity, connection 508 dimensionality 100	8.7×10^{-7}
	0683	Area of bankssts in the right hemisphere	9.3×10^{-7}
	2222	Functional connectivity, connection 4 dimensionality 100	2.8×10^{-6}
	0478	Volume of lateralorbitofrontal in the right hemisphere	2.9×10^{-6}

Abbreviations: Phenotype ID and brief description from Oxford Brain Imaging Genetics Server-BIG40; MO, diffusion tensor mode; FA, fractional anisotropy; MD, mean diffusivity; ICVF, intra-cellular volume fraction. *P*-value $< 1 \times 10^{-7.5}$ indicates the genome-wide significant association, and *P*-value $< 1 \times 10^{-5}$ indicates suggestive association.
